# Circulating ketone bodies and mortality in heart failure: a community cohort study

**DOI:** 10.3389/fcvm.2024.1293901

**Published:** 2024-01-24

**Authors:** Rebecca O. Oyetoro, Katherine M. Conners, Jungnam Joo, Sarah Turecamo, Maureen Sampson, Anna Wolska, Alan T. Remaley, James D. Otvos, Margery A. Connelly, Nicholas B. Larson, Suzette J. Bielinski, Maryam Hashemian, Joseph J. Shearer, Véronique L. Roger

**Affiliations:** ^1^ Heart Disease Phenomics Laboratory, Epidemiology and Community Health Branch, National Heart, Lung, and Blood Institute, National Institutes of Health, Bethesda, MD, United States; ^2^ Office of Biostatistics Research, National Heart, Lung, and Blood Institute, National Institutes of Health, Bethesda, MD, United States; ^3^ Department of Laboratory Medicine, Clinical Center, National Institutes of Health, Bethesda, MD, United States; ^4^ Lipoprotein Metabolism Laboratory, Translational Vascular Medicine Branch, National Heart, Lung, and Blood Institute, National Institutes of Health, Bethesda, MD, United States; ^5^ Labcorp, Morrisville, NC, United States; ^6^ Division of Clinical Trials and Biostatistics, Department of Quantitative Health Sciences, Mayo Clinic College of Medicine and Science, Rochester, MN, United States; ^7^ Division of Epidemiology, Department of Quantitative Health Sciences, Mayo Clinic College of Medicine and Science, Rochester, MN, United States

**Keywords:** ketone bodies, biomarkers, heart failure, mortality, epidemiology

## Abstract

**Background:**

The relationship between ketone bodies (KB) and mortality in patients with heart failure (HF) syndrome has not been well established.

**Objectives:**

The aim of this study is to assess the distribution of KB in HF, identify clinical correlates, and examine the associations between plasma KB and all-cause mortality in a population-based HF cohort.

**Methods:**

The plasma KB levels were measured by nuclear magnetic resonance spectroscopy. Multivariable linear regression was used to examine associations between clinical correlates and KB levels. Proportional hazard regression was employed to examine associations between KB (represented as both continuous and categorical variables) and mortality, with adjustment for several clinical covariates.

**Results:**

Among the 1,382 HF patients with KB measurements, the median (IQR) age was 78 (68, 84) and 52% were men. The median (IQR) KB was found to be 180 (134, 308) μM. Higher KB levels were associated with advanced HF (NYHA class III–IV) and higher NT-proBNP levels (both *P* < 0.001). The median follow-up was 13.9 years, and the 5-year mortality rate was 51.8% [95% confidence interval (CI): 49.1%–54.4%]. The risk of death increased when KB levels were higher (HR_high vs. low group_ 1.23; 95% CI: 1.05–1.44), independently of a validated clinical risk score. The association between higher KB and mortality differed by ejection fraction (EF) and was noticeably stronger among patients with preserved EF.

**Conclusions:**

Most patients with HF exhibited KB levels that were consistent with those found in healthy adults. Elevated levels of KB were observed in patients with advanced HF. Higher KB levels were found to be associated with an increased risk of death, particularly in patients with preserved EF.

## 1 Introduction

Heart failure (HF) is a complex clinical syndrome associated with metabolic alterations of several substrates, including glucose, amino acids, fatty acids, and ketone bodies (KB) (1). People with HF have higher circulating levels and myocardium utilization of KB (2–5). Increased KB oxidation may be a metabolic adaptation in HF (6), partly due to its higher phosphate-to-oxygen ratio compared with other substrates (7).

Animal and human studies have suggested KB supplementation as a potential therapeutic strategy in HF, postulating that KB could improve cardiac function by improving myocardial blood flow and cardiac remodeling, while reducing oxidative stress (8–11). However, due to the lack of standard clinical measurements of KB, there is a scarcity of human data in large cohorts of patients encompassing the entire HF syndrome, and their association with mortality in HF remains poorly understood. Addressing these gaps in knowledge in sufficiently large cohorts that allow for comprehensive correlative assessment and adjustment for key covariates is a prerequisite to promote supplementation interventions.

Nuclear magnetic resonance (NMR) spectroscopy now offers a high-throughput approach to measure circulating KB in epidemiologic studies, which will provide important insights into the associations between KB and HF prognosis. To do so, we measured the distribution of plasma KB in a large community cohort that includes individuals across the entire spectrum of the HF syndrome and examined the key clinical correlates and the association between KB and all-cause mortality.

## 2 Materials and methods

### 2.1 Patient population

This HF community cohort was assembled under the auspices of the Rochester Epidemiology Project (REP), a comprehensive record linkage system that captures clinical diagnoses, procedures, results, and outcomes in its catchment area (12, 13). Using natural language processing to analyze the content from electronic medical records, we assembled a community cohort consisting of all patients aged 20 years and older who were diagnosed with HF and lived in Olmsted, Dodge, or Fillmore Counties in Minnesota. The collection of data was previously published (14–16). This approach yielded 100% sensitivity compared with billing data, a reference method for case finding (17). Research nurses reviewed the records and validated the HF diagnosis using the Framingham criteria (18). Patients were approached in the hospital or after an outpatient encounter to provide written consent to participate in the study, including a blood draw, from 2 September 2003 to 16 June 2012. The Mayo Clinic and Olmsted Medical Center Institutional Review Boards approved this study.

### 2.2 Data collection

Nurse abstractors collected clinical information from both inpatient and outpatient records from all providers. Left ventricular ejection fraction (EF) was obtained from the closest available echocardiogram within 6 months prior to or 2 months following the date of enrollment. Body mass index (BMI) was calculated using weight (kilograms) from the last outpatient visit prior to enrollment divided by their earliest recorded adult height (meters) squared. The Meta-Analysis Global Group in Chronic HF (MAGGIC) score was calculated using sex, age, EF, systolic blood pressure, BMI, creatinine, New York Heart Association (NYHA) class, smoking status, diabetes, chronic obstructive pulmonary disease, HF diagnosis > 18 months ago, and the use of beta blocker, angiotensin-converting enzyme inhibitors, an/or angiotensin-receptor blockers (19). The MAGGIC score was originally derived to assess the mortality risk in patients with HF using the data collected from 39,000 HF patients, across the EF spectrum. Missing data were rare, and 10 multiple imputations by chain equations were performed to account for the missing components of the MAGGIC scores, including BMI (2.8%), NYHA class (0.4%), HF duration (0.1%), EF (1.8%), and creatinine (0.7%). N-terminal pro B-type natriuretic peptide (NT-proBNP) levels were measured at the NIDDK Clinical Laboratory Core using a Mesoscale multiplex assay, following the manufacturer’s instructions (https://www.mesoscale.com/en).

### 2.3 Biomarker measurements

NMR analyses of frozen EDTA plasma samples collected at enrollment were used to measure β-hydroxybutyrate (β-HB), acetoacetate (AcAc), and acetone performed on the high-throughput 400 MHz Vantera® Clinical Analyzer platform at the NHLBI Lipoprotein Metabolism Laboratory (Bethesda, MD). The three KB metabolites give rise to NMR signals that represent the basis of their quantification (20). For our analysis, the plasma levels of the three KB metabolites were summed to calculate the total KB as previously described (LabCorp, Morrisville, NC) (20).

### 2.4 Ascertainment of death

The patients were monitored until 31 March 2021, using data from REP. The REP obtains death date information from the healthcare providers that participate in the REP, from the State of Minnesota death certificates, and from the linkage of patient records to the National Death Index. The cause of death information is available from the Minnesota death certificates and from National Death Index. Patients who were still alive at the end of the follow-up period were censored on 31 March 2021, or the date of their last known healthcare contact, whichever was earlier.

### 2.5 Statistical analysis

Baseline characteristics were expressed as frequencies (percent) for categorical variables and as medians [interquartile range (IQR)] for continuous variables. Spearman correlation coefficients (*ρ*) were calculated across total KB and each KB metabolite (β-HB, AcAc, and acetone). KB levels were transformed into the natural logarithmic scale for all subsequent analyses and modeled both continuously (per 1 SD) and categorically. KB groups [low KB: ≤ 471.5 μM (*N* = 1,172); high KB: > 471.5 μM (*N* = 210)] were determined by a conditional inference tree method (ctree R Package), a recursive partitioning approach (21).

Multivariable linear regression was used to identify the clinical correlates independently associated with continuous KB levels. β-coefficients [95% confidence interval (CI)] were reported for each correlate.

The median follow-up time was calculated using the reverse Kaplan–Meier method (22). The survival by KB group was estimated using the Kaplan–Meier method and compared across groups by the log-rank test. Cox proportional hazards regression models were used to examine the association between KB and mortality. The models included (i) unadjusted, (ii) adjusted for age and sex, (iii) adjusted for MAGGIC score, and (iv) adjusted for MAGGIC and log2-transformed NT-proBNP (pg/ml). Stratified analyses were also performed by categorizing patients into reduced EF (< 50%) or preserved EF (≥ 50%).

Two-sided *p*-values of < 0.05 were considered statistically significant. The statistical analyses were performed using R version 3.6.2.

## 3 Results

### 3.1 Patient population

This HF cohort included 1,389 patients, with seven patients excluded due to the unavailability of their plasma samples, leaving 1,382 patients for analysis. The median (IQR) age for this analytic cohort was 78 (68, 84), and 52% were male (Table 1). Cardiometabolic risk factors were highly prevalent, including hypertension (91%), hyperlipidemia (85%), ischemic etiology (50%), and diabetes (36%). The median (IQR) MAGGIC score of the cohort was 24 (20, 29), and most patients were in NYHA class III–IV (69%). The distribution of patients with preserved EF (*N* = 785) and reduced EF (*N* = 597) was similar.

**Table 1:** Baseline clinical and laboratory characteristics.

Number of patients	1,382 (100)
Patient characteristics	
Age (years)	78 (68, 84)
Male	715 (52)
BMI (kg/m^2^)^a^	28 (25, 34)
Current smoker	144 (10)
Comorbidities	
Hypertension	1,261 (91)
Hyperlipidemia	1,171 (85)
Ischemic etiology	697 (50)
Diabetes	493 (36)
Chronic obstructive pulmonary disease	395 (29)
Heart failure characteristics	
NYHA class III–IV	956 (69)
EF (%)	
≥ 50	785 (57)
< 50	597 (43)
MAGGIC score	24 (20, 29)
HF duration ≤ 18 months	886 (64)
Medications	
Ace inhibitors	672 (49)
Beta blockers	1,047 (76)
Angiotensin-receptor blockers	222 (16)
Clinical laboratory measurements	
Creatinine (mg/dl)^a^	1.2 (1.0, 1.5)
NT-proBNP (pg/ml)	8,902 (4,198, 16,304)
NMR measurements	
KB measurements	
Total KB (μM)	180 (134, 308)
β-HB (μM)	102 (73, 183)
AcAc (μM)	41 (28, 71)
Acetone (μM)	37 (24, 63)

Data expressed as *n* (%) and median [interquartile range (IQR)]. Acetoacetate, AcAc; β-HB, beta-hydroxybutyrate; BMI, body mass index; KB, ketone bodies; MAGGIC, Meta-analysis Global Group in Chronic Heart Failure; NT-proBNP, N-terminal pro B-type natriuretic peptide; NYHA, New York Heart Association.

^a^ Imputed data.

**Figure 1:**
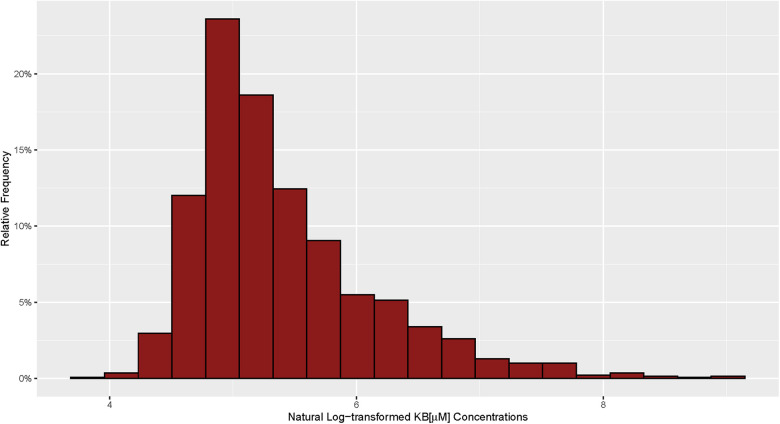
Distribution of circulating ketone bodies in 1,382 patients with HF.

The median (IQR) and mean (SD) KB concentration for the cohort were 180 μM (134, 308) and 332 μM (549), respectively, with a right skewed distribution (Figure 1). The Spearman's rank correlation of β-HB, AcAc, and acetone were highly correlated with plasma total KB (*ρ* = 0.92, 0.85, and 0.73, respectively) (Supplementary Table 1).

### 3.2 Correlates of KB in HF

Following the multivariable adjustment, NYHA class III–IV (β = 0.22, 95% CI: 0.10–0.33; *P* < 0.001) and NT-proBNP levels (β = 0.11, 95% CI: 0.07–0.14; *P* < 0.001) were independently and positively associated with KB levels (Table 2). No independent association between KB levels and age, sex, BMI, hyperlipidemia, ischemic etiology, EF, and diabetes was observed (*P* > 0.05).

**Table 2:** Multivariable linear regression of KB levels (per 1 SD) and clinical correlates.

Correlates	β	95% CI	*P*-value
Age (per 10 years)	−0.04	−0.09–0.01	0.09
Female sex	0.07	−0.03–0.18	0.18
BMI (per 5 kg/m^2^)	0.01	−0.03–0.05	0.78
Hyperlipidemia	−0.06	−0.21–0.09	0.44
Ischemic etiology	−0.01	−0.13–0.10	0.81
Diabetes	−0.01	−0.13–0.10	0.81
EF ≥50%	−0.01	−0.12–0.11	0.88
NYHA Class III–IV	0.22	0.10–0.33	<0.001
NT-proBNP (pg/ml)^b^	0.11	0.07–0.14	<0.001

Data are reported as β-coefficients (95% confidence interval). BMI, body mass index; KB, ketone bodies; NT-proBNP, N-terminal pro B-type natriuretic peptide; NYHA, New York Heart Association.

^b^ Log2-transformed.

### 3.3 KB and mortality

**Figure 2:**
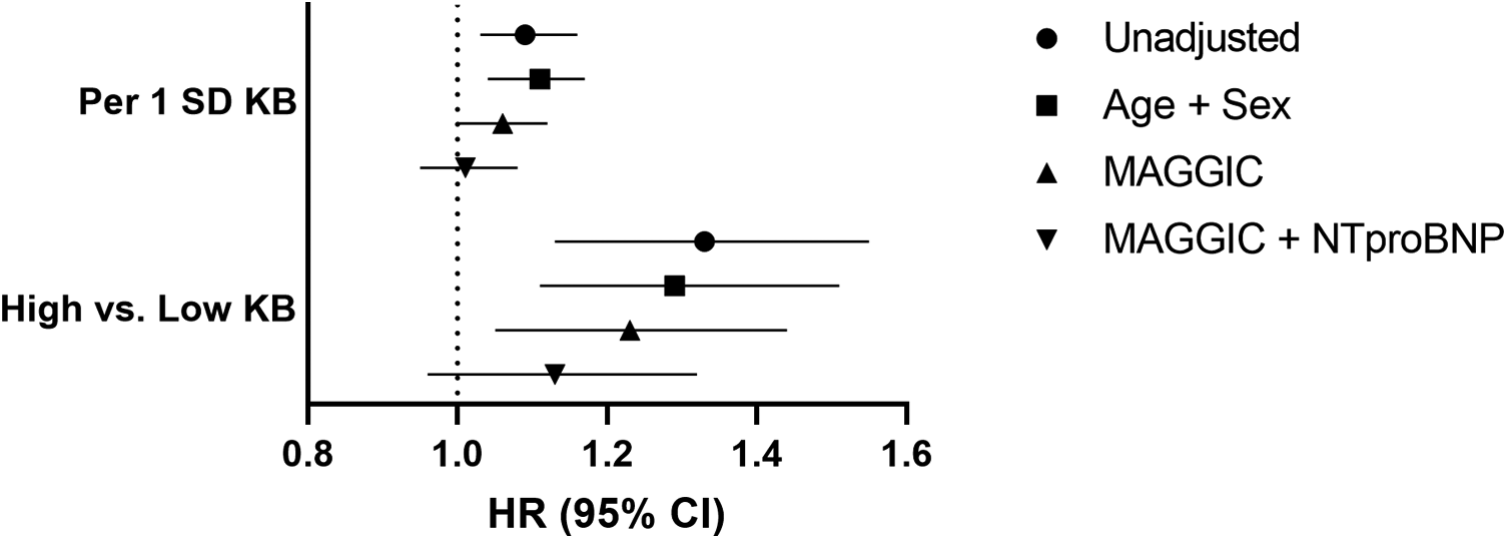
Ketone bodies and risk of mortality. Low KB: ≤ 471.5 μM (*N* = 1,172); High KB: > 471.5 μM (*N* = 210). Ketone bodies, KB; MAGGIC, Meta-analysis Global Group in Chronic Heart Failure; NT-proBNP, N-terminal pro B-type natriuretic peptide; standard deviation, SD.

The median (IQR) follow-up was 13.9 (11.5, 15.4) years. At the end of follow-up, 1,158 patients died with a 5-year mortality rate of 51.8% (95% CI: 49.1%–54.4%). A 1 SD increase in KB was associated with an increased risk of mortality (HR 1.09; 95% CI: 1.03–1.16). The increased risk remained even after further adjustment for the MAGGIC score (HR 1.06; 95% CI: 1.00–1.12), but it was attenuated after further adjustment for NT-proBNP (HR 1.01; 95% CI 0.95–1.08) (Figure 2).

**Figure 3:**
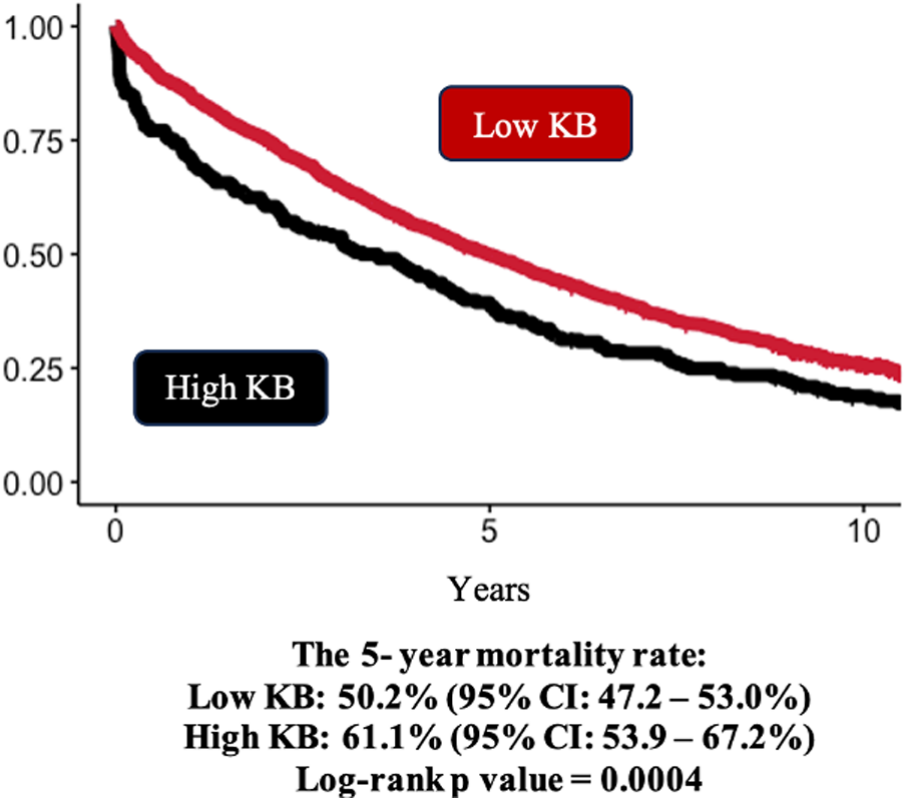
Kaplan–Meier survival curves by ketone bodies groups. Kaplan–Meier estimates and log-rank *P*-values of all-cause mortality. Low KB: ≤ 471.5 μM (*N* = 1,172); High KB: > 471.5 μM (*N* = 210). Patients in the high KB group had higher rates of mortality compared with those in the low KB group.

**Figure 4:**
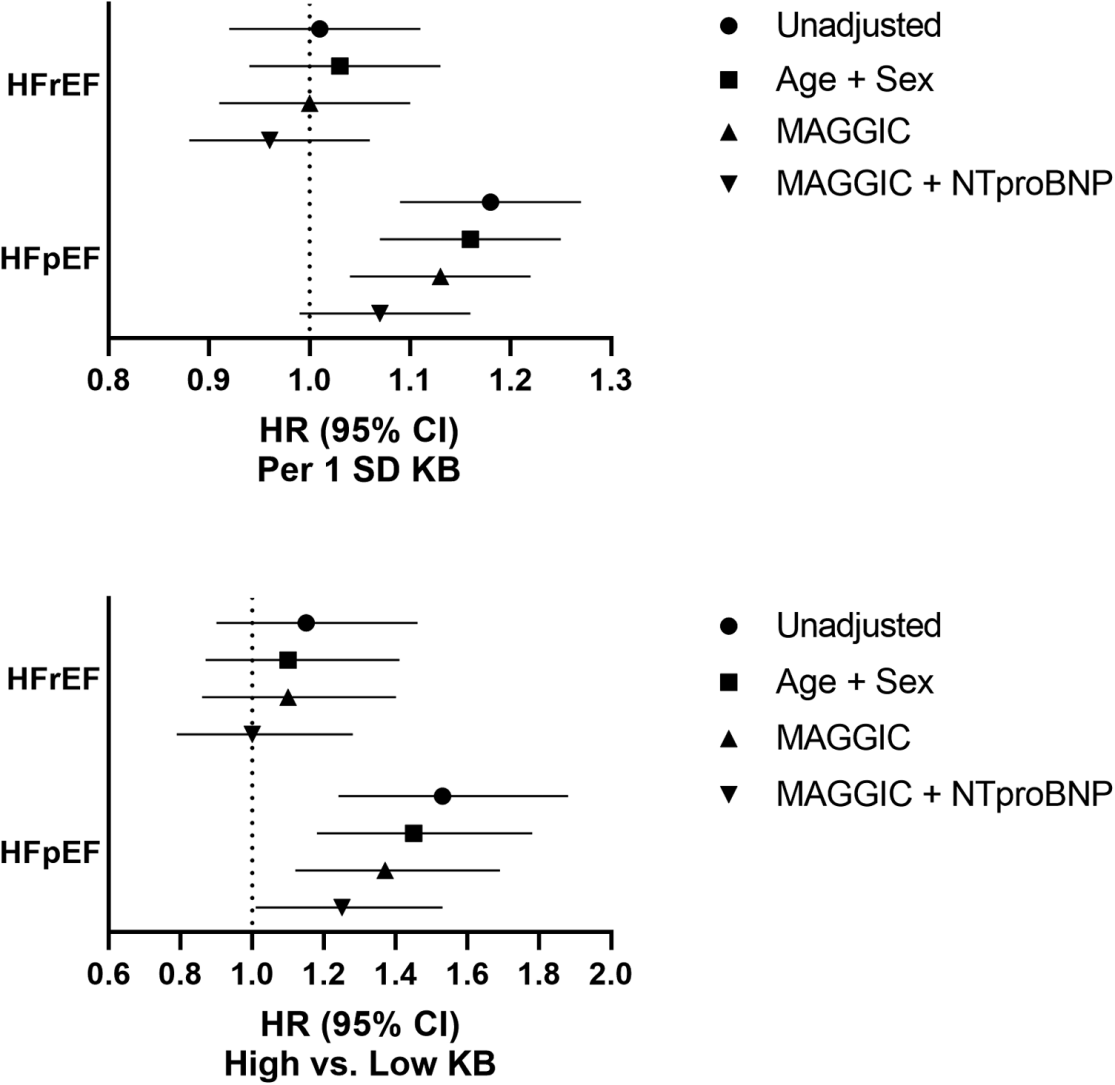
Ketone bodies and risk of mortality stratified by ejection fraction category. Low KB: ≤ 471.5 μM (*N* = 1,172); High KB: > 471.5 μM (*N* = 210). Ketone bodies, KB; MAGGIC, Meta-analysis Global Group in Chronic Heart Failure; NT-proBNP, N-terminal pro B-type natriuretic peptide; standard deviation, SD.

When analyzing KB categorically, the 5-year mortality rate in the high KB group was 61.1% (95% CI: 53.9%–67.2%), compared with 50.2% (95% CI: 47.2%–53.0%) in the low KB group (Figure 3). Similar to the continuous results, the patients in the high KB group were at greater risk of mortality compared with the patients in the low KB group (HR 1.33; 95% CI: 1.13–1.55). An increased risk of mortality remained even after further adjustment for the MAGGIC score (HR 1.23; 95% CI: 1.05–1.44), but it was attenuated after further adjustment for NT-proBNP (HR 1.13; 95% CI 0.96–1.32) (Figure 2). The overall association between mortality and KB remained consistent regardless of the modelling strategy (KB modeled continuously or categorically). However, the association between KB and mortality differed by the EF group, with a higher risk of mortality observed in patients with preserved EF but not in patients with reduced EF (Figure 4).

## 4 Discussion

Herein, we report on the distribution, clinical correlates, and association with mortality of plasma KB in a community cohort of HF patients. We revealed that, in this population, which represents the consecutive experience of a geographically defined community with HF, most KB levels were similar to values obtained in healthy adults (20). Higher KB levels were associated with higher NYHA class and higher NT-proBNP levels. Finally, while the elevated KB levels increased the risk of death modestly across the entire spectrum of the HF syndrome, the association differed by EF and was particularly strong among HF patients with preserved EF. The association was independent of the MAGGIC score and only partially attenuated after further adjustment for NT-proBNP, indicating that higher KB levels may not be protective in HF.

### 4.1 Distribution of KB in HF

To the best of our knowledge, no consensus reference values for KB have been established in HF. Prior reports indicated that patients with HF had elevated KB levels compared with patients without HF (2, 3, 5). One study of 45 patients with congestive HF reported KB levels ranging from 44 to 952 μM (2). In this HF community cohort, the median and mean KB concentrations were 180 μM and 332 μM, respectively. In non-fasting healthy adults, the KB levels, measured using the same method as our study, ranged between 100 and 600 μM and increased to 1,000 μM following prolonged fasting or exercise (20). As a point of reference, in diabetes or alcohol ketoacidosis, the KB levels can range between 6,000 and 20,000 μM (20, 23, 24). These findings suggest that most patients drawn from an HF community cohort have KB levels that fall in the expected range in healthy adults.

### 4.2 Correlates of KB in HF

Herein, we found that higher NYHA class and higher NT-proBNP were associated with higher KB levels, consistent with the postulate that KB levels increase with HF severity to maintain adequate cardiac function (2, 5, 25). Indeed, in a study of non-ischemic HF patients, exhaled acetone levels, which were correlated with blood KB levels, were associated with higher NYHA class and higher BNP (26). In a study of 45 patients with HF, higher KB levels were correlated with a lower EF (2), while another study of 46 patients with HF with reduced EF found higher levels of acetone among HF patients compared with controls (27). However, we observed no cross-sectional association between KB and EF categories after controlling for several potential confounding factors, indicating that our findings surrounding KB correlates apply to the entire spectrum of the HF syndrome.

The relationship between natriuretic peptides and KB in patients with HF is not entirely understood. Several studies have shown an association between natriuretic peptides and lipolysis (28), which can result in the production of KB. The stimulation of lipolysis by natriuretic peptides has been suggested as a potential mechanism of cachexia in patients with advanced HF (29). We found a strong positive association between KB levels and NT-proBNP. In a Dutch population cohort of 6,134 participants, β-HB concentrations were associated with higher NT-proBNP at baseline and an increased risk of HF with reduced EF (30). Several studies reported that elevated KB levels were positively associated with other natriuretic peptides, including BNP (31) and pro-atrial natriuretic peptide (2). These findings suggest that the interplay between KB and natriuretic peptides may be associated with HF severity. Further work is needed to understand the molecular underpinnings of this potential association.

Diabetes increases the risk of mortality in HF (32). Previous studies have shown higher KB levels may be associated with diabetes overall (20) and among patients with advanced HF and diabetes (33). In this cohort, we observed no relationship between diabetes and KB levels. Several reasons could explain the difference in findings, including large differences in the characteristics of the study population (age, race, ethnicity, disease status) and study design (clinical cohort vs. population-based study). However, in light of the recent evidence suggesting that sodium-glucose transporter 2 inhibitors, a drug class originally designed to treat diabetes, reduce the risk of HF mortality (34) and also increase KB levels (35), further efforts are needed to unravel the complex interplay between diabetes, KBs, and HF mortality.

### 4.3 KB and mortality

There is limited evidence on the association between circulating KB levels and HF mortality. A study of 615 HF patients found that patients with high AcAc concentrations (≥ 35 μM) were at a small but detectable increased risk of all-cause mortality (36). Several studies have found an association between higher breath acetone levels and HF (26, 27, 33, 37). Elevated levels of exhaled breath acetone have been reported to be an independent predictor of mortality in HF with reduced EF (25, 38). Using NMR spectroscopy, a population-based, multi-ethnic study including 6,796 subjects from the United States found that higher levels of β-HB and AcAc were associated with cardiovascular events and mortality (39). Another study of older adults found that higher KB levels were associated with both incident HF and all-cause mortality, independent of metabolic and cardiovascular risk factors (40). Although these results are aligned with our finding and support that KB may be associated with a poor prognosis in HF, it remains unclear whether increased KB metabolism is a cause or a consequence of HF (41). Hence, further studies are needed to evaluate a possible causal relationship between KB levels and mortality.

Although we observed no cross-sectional association between KB levels and EF categories, the stratified analyses showed that the association between KB levels and mortality differed by EF and was noticeably stronger if the EF was preserved. Conceptually, KB utilization occurs in HF regardless of EF (7); however, its utilization may differ by EF in patients with reduced EF using nearly three times as much KB compared with patients with preserved EF (42). The differences in KB utilization in the myocardium in the absence of any apparent association with circulating KB levels might provide a plausible biological mechanism for the observed differences in mortality by EF. Additional research is required in study populations that comprise the entire spectrum of HF with mortality data to further test this hypothesis.

While higher KB levels remained associated with increased mortality after adjustment for MAGGIC in our study, further adjustment for NT-proBNP attenuated the association between KB and mortality. This may reflect overadjustment of our model as the MAGGIC risk score uses 13 routinely collected long-established predictors of mortality in HF, specifically, NYHA classification and EF, which are associated with NT-proBNP (19, 43, 44).

KB supplementation has been suggested as a therapeutic intervention in human and animal studies (8, 45–47), arguing for protective effects of KB in patients with HF (7). Some studies reported that an increase in circulating KB levels is associated with a favorable increase in myocardial KB utilization in HF (4, 5, 48). In clinical studies, it has been posited that ketone supplementation may prevent pathological remodeling, improve cardiac work, and reduce cardiovascular risk factors in HF patients (10, 11, 49). A small, randomized crossover study of 16 patients with chronic HF and reduced EF reported a dose–response association with β-HB infusion and oxygen consumption without affecting myocardial external energy efficiency (11). However, there is clearly insufficient evidence regarding the therapeutic potential of KB in HF. Our findings of a modest but significant increase in the risk of death associated with higher levels of plasma KB do not support the notion that higher levels of KB would be protective in HF, but we cannot rule out the possibility that higher levels of KB may be an adaptive mechanism of advanced HF.

### 4.4 KB and metabolic alterations in HF

For over half a century, researchers have postulated that metabolic alterations may play a role in HF and cardiac cachexia, including KB (50). Evidence suggests that cardiac metabolism can impact both the structure and function of the heart (51). However, our understanding of the biological underpinnings of the association between KB and HF are complicated by the fact that several metabolic alterations are likely occurring simultaneously as is evident by multiple reports showing an association between KB and triglyceride levels (52, 53). Further work, probably involving more comprehensive metabolomics, may be needed to unravel the complex interplay between these metabolic alterations and the potential functional consequences of altered KB levels and mortality in HF.

### 4.5 Limitations and strengths

The majority of our cohort consisted of individuals who identified as non-Hispanic White, which potentially limits the generalizability of our findings to more ethnically and racially diverse populations. Fasting was not required to be enrolled in the study; however, most patients had NMR-based glucose levels consistent with fasting. Comparing results across published studies is challenging due to study heterogeneity, varying sample sizes, and differences in KB measurements. While we observed an association between KB and mortality, comorbidities and polypharmacy are highly prevalent in patients with HF. While we relied on the MAGGIC score as a key adjustment variable, this score does not fully account for all the possible prognostic indicators in HF such that we cannot rule out potential residual confounding.

Our study had several strengths. To the best of our knowledge, this study is among the first to characterize the distribution of KB in HF, establish clinical correlates of KB elevation, and assess the association between KB levels and mortality in a population-based cohort comprised of the entire spectrum of HF. By studying a community cohort, we optimize the applicability of our results to routine clinical practice. The long-term follow-up and rich clinical dataset available through the Rochester Epidemiology Project allowed us to control for several key covariates and potential confounders of the association between KB and mortality.

### 4.6 Conclusion

In a cohort of individuals with HF, the distribution of KB levels is primarily within the expected ranges for healthy individuals. These KB levels are correlated with indicators of HF severity and are independently associated with mortality in a population-based cohort comprised of the entire spectrum of HF, in particular among patients with preserved EF.

## Data Availability

The datasets generated for this study can be made available upon reasonable request to the corresponding author. Requests to access the datasets should be directed to Véronique L. Roger, veronique.roger@nih.gov.
