# Supplementary material for: Circulating ketone bodies and mortality in heart failure: a community cohort study
Source: Front Cardiovasc Med. 2024 Jan 24;11:1293901. doi: 10.3389/fcvm.2024.1293901 (PMC10847221; doi:10.3389/fcvm.2024.1293901)
Supplement: Supplementary file 1 [file Table1.docx]

# SUPPLEMENTAL TABLES

**Supplemental Table 1. Spearman correlation coefficients between total ketone bodies (KB) and individual ketone body metabolites.**

|  | **Total KB** | **β-HB** | **AcAc** | **Acetone** |
| --- | --- | --- | --- | --- |
| **Total KB** | 1.00 |  |  |  |
| **β-HB** | 0.92 | 1.00 |  |  |
| **AcAc** | 0.85 | 0.75 | 1.00 |  |
| **Acetone** | 0.73 | 0.51 | 0.55 | 1.00 |

Beta-hydroxybutyrate= β-HB; Acetoacetate = AcAc
